# Maintaining the Partnership Between a Tribal Breast and Cervical Cancer Program and a University-Based Cancer Prevention Center During COVID-19 Lock-Down Restrictions-A Case Study

**DOI:** 10.3389/fpubh.2022.902253

**Published:** 2022-07-13

**Authors:** Nicolette I. Teufel-Shone, Carol Goldtooth-Begay, Andria B. Begay, Ashley Lazaro, Janet Yellowhair, Rolanda Todecheenie, Delila Begay, Darlene Singer, Curtis Briscoe

**Affiliations:** ^1^Center for Health Equity Research, Department of Health Sciences, Northern Arizona University, Flagstaff, AZ, United States; ^2^School of Medicine and Health Sciences, University of North Dakota, Grand Forks, ND, United States; ^3^Navajo Nation Breast and Cervical Cancer Prevention Program, Navajo Nation Department of Health, Window Rock, AZ, United States

**Keywords:** COVID-19, podcast, partnership building, Native Americans, Navajo Nation

## Abstract

To inform women of the Navajo Nation of safety measures implemented to minimize COVID-19 virus exposure during screening and treatment procedures at Navajo Nation based health care facilities, the Navajo Nation Breast and Cervical Cancer Prevention Program (NNBCCPP) and the University-based Partnership for Native American Cancer Prevention Program (NACP) collaborated to develop a podcast to describe the continued availability of services. During the COVID-19 pandemic, women of all ages and ethnicities in the US needing breast and cervical cancer prevention screenings and treatment, have been hesitant to seek services given the advice to avoid crowded spaces and maintain physical distancing. Epidemiological trends indicate that proactive, intensive strategies are needed in Native American communities for early detection and treatment to support early cancer diagnosis and improve cancer survival. The NNBCCPP and Northern Arizona University (NAU) through the National Institute of Health's National Cancer Institute funded NACP had a nascent partnership prior to the onset of COVID-19 pandemic. This partnership relied on face-to-face interaction to allow for informal social interaction, facilitate clear communication and support continued trust building. To adhere to federal, state and tribal recommendations to minimize gatherings and to stay in-place to minimize the spread of the virus, the Navajo Nation and NAU restricted, and in most cases would not approve employee travel for partnership meetings. The plans to develop a podcast necessitated bringing additional members into the collaboration who were unfamiliar to the original partners and due to travel restrictions, required all interactions to be remote. This expanded group met virtually to develop a script, record and edit the podcast. More importantly, group members had to build and maintain trust over months of communicating *via* a teleconference video platform. This collaborative addressed challenges related to unstable Internet connections and periodic stay-at-home policies; thus, these emerging partners had to modify social and professional communication to respect and accommodate the stress and uncertain circumstances created by the pandemic on the citizens and employees of Navajo Nation. This case study describes strategies used to maintain and respect all members of the partnership.

## Introduction

In many close-knit communities, trust and credibility are conveyed in face-to-face interactions (1, 2). In Native American communities often composed of individuals who have known each other all their lives, community-based education has relied heavily on personal, direct interactions often with educators who are from the community and/or have worked in the community for a number of years. Those educational interactions occur through health fairs, household visits and community gatherings (3–6). In the early months and continued response to the COVID-19 pandemic, many tribal councils acted to protect their citizens living on their reservations, by issuing executive orders, that restrict travel out of the home to reduce the risk of exposure to non-household members (7, 8). Locally, this restriction is known as Lock-down.

Adapting to these restrictions, the Navajo Nation Breast and Cervical Cancer Prevention Program (NNBCCPP) and the University-based Partnership for Native American Cancer Prevention Program (NACP) collaborated to develop a podcast to inform women of the Navajo Nation of the continued availability of services and the safety measures implemented to minimize COVID-19 virus exposure during screening and treatment procedures at Navajo Nation based health care facilities.

The need for cancer-related education about healthy lifestyles and regular health screenings has not stopped during the pandemic (9). Recent epidemiological trends indicate that proactive, intensive strategies are needed in Native American communities for prevention, early detection and treatment to support early cancer diagnosis and improve cancer survival (10–12). Lock-down poses a challenge to the ways cancer education has been provided in the past in Native American communities. Lay and professional health educators can no longer organize health fairs or community gatherings or conduct home visits safely under the threat of the ever-present COVID-19 virus. Yet, many Native communities have local radio stations and citizens often are familiar with podcasts, used pre-COVID during long-distance trips across or off large, rural reservations, when radio signals are unstable or jump from station to station. Radio programming and podcast development offer an opportunity to reach community members to provide cancer-related education messages and explain modifications in local cancer screening and treatment services.

## Background

In September 2020, the Outreach Core of the Native American Cancer Prevention Partnership (NACP), a partnership between University of Arizona Cancer Center and Northern Arizona University and a center funded through the National Institutes of Health-National Cancer Institute's (NIH-NCI) U54 mechanism Partnership to Advance Cancer Health Equity (PACHE), began to discuss ways to support Native American communities in cancer-related education during Lock-down restrictions. The Outreach Core reached out to the Navajo Nation Breast and Cervical Cancer Prevention Program (NNBCCPP), a Center for Disease Control and Prevention (CDC) cooperative grant agreement, to explore alternative strategies for cancer-related education during Lock-down. Some members of the NACP and NNBCCPP teams had collaborated previously on cancer education efforts, so a nascent relationship had been established. NACP Outreach had provided educational materials to NNBCCPP to share at health fairs and local conferences and the NNBCCPP Director had offered suggestions to NACP about ways to reach Navajo Nation citizens in health promotion efforts. Yet, some team members in both programs were new to the collaboration and had not established a rapport.

A NACP Outreach team member who had worked with NNBCCPP previously, contacted the Program Director to request a virtual meeting to discuss continued collaboration during COVID- 19 restrictions. In this meeting, the group first discussed the impact of COVID-19 and Lock- down on cancer prevention and care within the Navajo Nation. The NNBCCPP Director described the program's pre-COVID strategies to reach and educate clients and to schedule cancer screenings. Due to COVID-related travel restrictions, the NNBCCPP case managers could no longer rely on face-to-face interactions and had to establish new ways of connecting with community members and providing education and screening services. NACP and NNBCCPP discussed Navajo Nation citizens' reliance on the tribal radio stations for information during Lock-down. The teams discussed a podcast series to share health information with Native American listeners and proposed one podcast with NNBCCPP to explain its services and safety precautions. The NNBCCP Director was receptive but wanted to talk to his team before committing to the idea. If they were interested, future meetings would include them as well.

Once contacted by their Director, the NNBCCPP case managers were interested in developing and speaking in the podcast, so bimonthly meetings were scheduled. In successive virtual meetings, the NACP and NNBCCPP teams began the process of developing the format and script of the episode. As these developmental meetings continued, members of both teams realized they were adapting to a new form of relationship building. Both teams had new members who had not worked together previously, and the virtual format was not conducive to casual, non-work- related conversation that would have established a degree of familiarity. Yet, this changing community-university collaboration needed to establish trust, credibility and accountability not only among themselves but also with the citizens of the Navajo Nation.

## Context and Partners

The Navajo Nation or Diné Bikéyah is a federally recognized tribe with a land base that spans over 27,000 square miles (approximately the size of West Virginia), extending into Arizona, New Mexico, Colorado and Utah. Recently, the Navajo Nation announced that tribal enrollment surpassed 400,000 citizens (13). A little less than half of the population reside on the Nation (14). The Navajo Nation is a rural area having a population density of 6.33 persons per square mile; the average population density for the U.S. is approximately 345 persons per square mile (15). Subsequently, residents of the Navajo Nation regularly travel long distances (>40 miles one way) to purchase food and supplies and to receive health care services.

### Navajo Nation Breast and Cervical Cancer Prevention Program

NNBCCPP was funded initially in 1996 through a cooperative agreement with the CDC to implement a program to provide breast and cervical cancer screening services to uninsured and underinsured women and use evidence-based strategies to reduce structural barriers to screening within health systems (16). This funding is based on the “Breast and Cervical Cancer Mortality Prevention Act of 1990 (Public Law 101-354).” The program's mission statement is to reduce breast and cervical cancers by engaging communities and partners to promote, increase, and improve the quality of health outcomes. The NNBCCPP goal is to improve the quality of life on the Navajo Nation through the prevention of breast and cervical cancers. Program intervention strategies are to provide education, screening, case management, and cancer patient navigation services.

**Figure 1:**
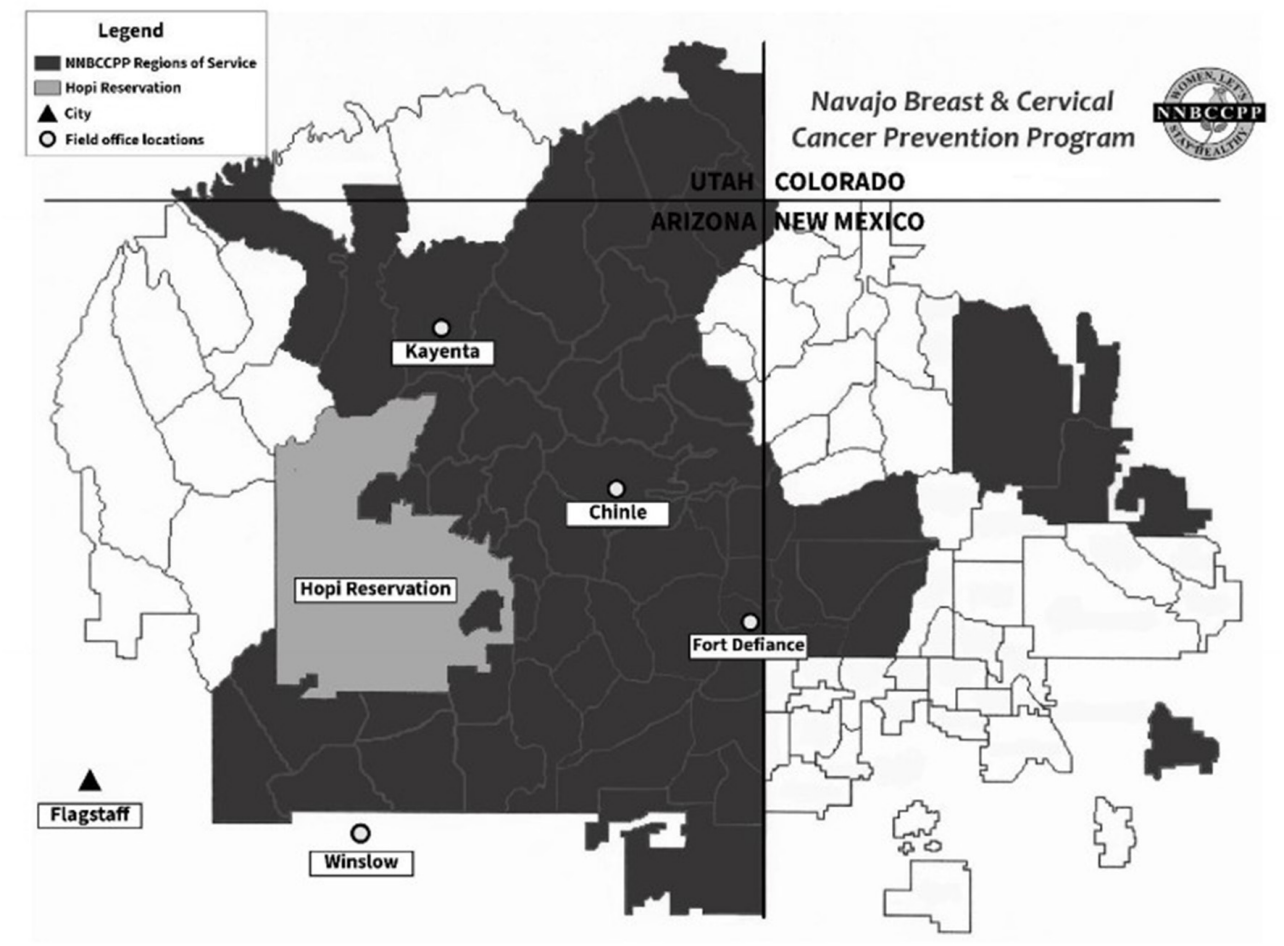
Service area of the Navajo Nation Breast and Cervical Cancer Prevention Program (19).

The NNBCCPP operates under the Navajo Nation Department of Health and covers Arizona and certain New Mexico portions of the Navajo Nation (Figure 1). Health care services on the Navajo Nation are regionalized into eight services areas by the Indian Health Service. The NNBCCPP provides services and contracts cancer screening to four service units in Arizona and portions of two in New Mexico. The priority population is low income and uninsured or underinsured women, ages 40–64 years, who have never received breast screening services and women, ages 21–64 years, who are eligible for cervical screening services. The Program has four field office in Arizona, in Chinle, Fort Defiance (Tséhootsooí), Kayenta, and Winslow. The Program extends cancer education to the entire Navajo Nation through direct intervention and partnerships with twelve clinics, health centers and hospitals, state-level cancer programs and community organizations serving Navajo Nation citizens in northern Arizona off the Navajo Nation, i.e., Flagstaff, AZ, Sanders, AZ and Bloomfield, NM. The Program expands mammography services through a mobile contractor to serve the rural and remote health care facilities on Navajo Nation.

The NBCCPP five-year summary for July 2014–June 2019 reported serving 4,737 women. Of this total, 1,418 women received cervical cancer screening and diagnostic services, and 3,795 women received breast cancer screening and diagnostic services; some women received both services. Approximately, 950 women were screened per year (2015–2019) (17).

NNBCCPP data shows that in 2017–2019, 8% of all mammography results were abnormal. In the latest data posted in 2021, the percent of abnormal mammography results has increased to over 11% (18). The NNBCCPP Director strategies to address this increase are to: (1) expand mobile mammography to previously unserved locations, and (2) recruit never screened women who may not know about cancer screening or did not know they had access.

### Native American Cancer Prevention Partnership

For the purposes of the podcast development, NACP Outreach team had five members: One faculty member (non-Native), one project coordinator (Navajo), one PhD student (Navajo) and two MPH students (one Navajo, one non-Native). The faculty member and the project coordinator knew the NNBCCPP Director but had not connected in person for more than a year. The project coordinator had worked with NNBCCPP for more than 5 years and knew the Director and two of the case managers. The student members of the Outreach team did not know the NNBCCPP team members.

## Methods: Developing the Podcast

### Preparation

As the producers of the proposed podcast series, the NACP Outreach team met to pool their knowledge of podcasts in general and refine the intention of the series. The team, recognizing their limited understanding of the construction of podcasts, decided to listen to several podcasts developed for Native American audiences and identify appealing elements (e.g., music, format). In addition, the team sought further consultation and advice from the Community Engagement Core (CEC) of NAU's NIH-National Institute of Minority Health and Health Disparities (NIH-NIMHD) funded Southwest Health Equity Research Collaborative (SHERC) (U54MD012388) that had started to develop a health equity podcast series, *Fairness First* (20). During this meeting, the SHERC team discussed its successes and difficulties, provided recommendations for the duration of each episode, the need for theme music to tie together the episodes, and suggested transcribing the audio recorded for the podcast to facilitate future editing. The SHERC team also recommended contacting NAU's Media Innovations Center in the School of Communications to assist in the development process.

The NACP Outreach Core met with the NAU Media Innovations Center Director to request guidance in this podcast venture. An outcome of this meeting was the decision to hire a current NAU media student to join the podcast development team.

### Planning and Connecting

From October 2020 to January 2021, the NACP team met weekly to develop the podcast plan, outline the script, and identify interviewees based on information, ideas and feedback received from the bi-monthly meeting with NNBCCPP staff. In the joint NACP- NNBCCPP bimonthly meetings, the NACP team would share preliminary materials. Although co-drafting materials would have been ideal, the NNBCCPP team members were quite busy working with clients and establishing safe service protocols and the NACP team members had time as many of their community outreach activities had been discontinued, as the university was not approving employee travel. In each meeting, the NNBCCPP team members were reminded the materials were draft, easily modified and intended to showcase NNBCCPP's work. The NNBCCP Director offered several changes, stating that he wanted to start by explaining the intent of the overall program followed by a description of the safety precautions instituted by the staff to reduce COVID-19 exposure, especially when visiting health care facilities for cancer screening or treatment.

All meetings were virtual and arranged to allow both phone and Zoom^tm^ connections. For the first 5 months of meetings, no NNBCCPP team member had a computer with a camera, so they would join via Zoom^tm^ without a picture or via a telephone. Oftentimes their internet connection was inadequate to support a Zoom^tm^ connection; the telephone offered a more stable connection. Members of both teams struggled to put a name to a voice, since video capabilities were limited.

In first 4 months of meetings, progress on the podcast development was slow but on-going conversations led to an understanding and appreciation for respective work environments. The NNBCCPP team members' work environment changed from home, office and car as they searched for internet connectivity and complied with quarantine requirements of Lock-down. For NNBCCPP team, technological conditions ranged from limited to no internet connectivity; personal cell phones were used when coverage was available. Their work conditions varied from taking care of and assisting children who were completing schoolwork virtually, assuring their own household food supplies were sufficient since now children who pre-COVID would eat at school, were now eating all meals at home, to addressing family health responsibilities linked to COVID-19 related illness.

Committed to their work responsibilities even during Lock-down, NNBCCPP case managers were reconnecting with patients by phone to discuss and schedule screening and treatment, and with health care facilities to arrange patient appointments and follow up on the patient health status.

### Implementing Podcast Plans

In December 2020, the NACP team invited a Navajo NAU project coordinator who co-led the SHERC podcast development, to meet the NNBCCPP team and potentially to serve as the podcast host. She had previous podcast and broadcast experience. She developed a rapport with the NNBCCPP staff and both teams agree she would be an appropriate podcast host for this episode.

### Interviews

*Four* interviews with the NNBCCPP staff were recorded over Zoom^tm^. This step required coordinating when the host and the interviewees were both available to record the jointly developed script. The NNBCCPP staff also recorded the interview on an additional device (cell phone or handheld recorder) in case the connection was lost or unstable during the Zoom^tm^ session.

The NACP team divided the interviews into three recording sessions, based on NNBCCPP staff availability. At this time, Navajo Nation had strict public health orders due to the COVID-19 pandemic and the NNBCCPP staff were working from home. Since several NNBCCP staff did not have internet access in their homes, they proceeded with their interviews using the Zoom^tm^ call-in feature. The NNBCCPR Director recorded his session first, followed by a single case manager and then two case managers were interviewed together.

The primary content of the podcast was edited versions of the recorded statements of the NNBCCPP Director and staff, all Navajo citizens. This approach assured the descriptions and essentially the voice of the podcast was culturally appropriate and centered.

## Partnership Experience

The NNBCCPP and NACP Outreach teams had to leverage the limited social connections established before the pandemic and navigate virtual challenges as they worked to develop a podcast episode during the COVID-19 pandemic. As has been widely reported in the media (21), the COVID-19 2020-2021 pandemic had a significant impact on the Navajo Nation, yielding a high infection rate, loss of life, food insecurity and obstacles for tribal services to maintain normal activities. With ongoing travel restrictions and concerns for health and safety, the NACP and NNBCCPP teams had to shift their in-person approaches to collaboration to connecting with partners virtually. Given the impersonal nature of the virtual connections, all participants began to realize they needed to take time in the virtual meetings to ask about work and if appropriate life circumstances, so all partners were aware on-going challenges and discussions of next steps did not add stress to team members' family and work responsibilities.

### Challenges

The NNBCCP staff has experienced a cycle of Lock-down orders mandated by the executive order of the Navajo Nation. The requirement to stay home impacted their ability to communicate as they are away from their offices, computers, and stable internet connections.

Yet, they continued to join the planning meetings and recording sessions, a reflection of their commitment and persistent in ensuring information about their program is disseminated to Navajo citizens. The NACP team has been conscious of the stress created by the Lock-down orders and faced feelings of being insensitive to their partners. The NACP and NNBCCPP teams acknowledged the loss of face-to-face communication and the impact of the ability to develop rapport.

While the NACP and NNBCCPP teams were able to use Zoom^tm^, challenges with an unstable internet connection, dropped calls and feedback noise made some of the recordings have an echo or were inaudible. NNBCCPP team members who did not have internet connection during Lock- down, had to use personal cellphones or landlines. Others did not have access to computers or laptops that had cameras, so many meetings were conducted without face-to-face interaction. For NACP and NNBCCPP team members who did not have previous familiarity with individuals on the call, it was not possible to put a name to a face and a voice.

For the Navajo team members with both NACP and NNBCCP, the virtual environment deterred the common Navajo custom of establishing kinship through humor and teasing which would have taken place in an in-person setting. The stated time schedule of the virtual meetings led to the team members not waiting to waste time, to start the meeting with an agenda and “skip” casual social interaction, common in a face-to-face meeting. Similarly, NACP added confusion by adding the Communications student and the SHERC host. NNBCCPP team members could not see the screen but only make note of an additional name and voice.

### Benefits

Despite COVID-19 pandemic restrictions and limitation, NACP and NNBCCPP built a relationship grounded in their shared commitment to serve the people. Their success was directly related to their persistence and dedication as they adapted to virtual platforms. Embracing the virtual reality imposed by pandemic restriction elevated project partners to be sensitive to and respectful of different and sometimes changing environments and resources.

The development of the podcast was a first-time experience for both NACP and NNBCCPP team members, allowing partners to co-learn the processes of recording and interviewing on Zoom^tm^. The teams were creative in thinking about how to broadcast information about the NNBCCPP services and safety precautions, as they could not rely on flyers and brochures, a common pre- COVID-19 approach to dissemination. Together, they worked through the podcast development.

## Contribution to Community-University Partnership Building

Communication and building on community partnerships during a pandemic in innovative ways is imperative, especially for community education, which informs the public and community members about public health issues. Looking at alternative mediums of communication, other than face-to-face community health education, social media platforms and podcasts are a resource to disseminate information.

As project partners, the NACP and NNBCCPP team members recognized that the COVID-19 pandemic created hindrances in everyday life and stressful work conditions. Acknowledging the changes that have come with the pandemic reinforced the need to collaborate, allowing NACP and NNBCCPP to advance the partnership and gain new skills in reaching their Navajo audience. NACP and NNBCCPP adapted to the new circumstances and made modifications throughout the podcast process, requesting additional time for communication to overcome the absence of face- to-face and building trust over Zoom^tm^ and phone, even for those new to the partnership.

NACP students gained knowledge and detailed information about the NNBCCPP program and specific strategies to promote cancer screening, create safe service protocols and the continuance of one-on-one communication between case managers and clients by phone.

In their willing participation with the podcast development, NNBCCPP team members learned the process of creating and developing a podcast, i.e., content and script development, revisions, host and interviewee selections, scheduled recording sessions, audio editing and social media dissemination.

Overall, the process made NACP and NNBCCPP team members recognized their resilience and the value of communicating and developing an effective partnership through a virtual venue.

Team members appreciated each other's persistence, commitment and contributions to using alternative communication strategies. Together, they were able to establish relationality, trust and credibility among themselves and with their audience.

## Recommendations for Community-University Partnerships Working Remotely

The foundation of partnership is relationality. Relationality or the ways individuals are connected, encompasses the processes of building effective communication and trust, and agreeing on expectations related to roles and accountability. Drawing on our face-to-face and more recently remote work, partnerships should be purposeful in: (1) establishing an ongoing communication strategy, both system and frequency, with which all partners are comfortable and can adhere; (2) building trust by being respectful of collaborators' perspectives and suggestions on how to accomplish a joint task and of each partners' contribution to the outcome; and (3) co-creating and periodically reviewing and revising individual responsibilities, and timelines and deadlines for established tasks. Flexibility and empathy in any collaboration contribute to a congenial, safe workspace but when non-verbal communication, e.g., body language and facial expressions, are difficult to discern in remote communication, these recommendations can enhance comradery and support open communication to yield an effective partnership.

## Data Availability Statement

The original contributions presented in the study are included in the article/supplementary material, further inquiries can be directed to the corresponding author.

## Author Contributions

Conceptualization: NT-S, CG-B, and AB. Methodology and data acquisition: AL, JY, RT, DB, DS, and CB. Writing—original draft preparation: NT-S, CG-B, AB, AL, JY, and CB. Writing—review and editing: NT-S, CG-B, AB, and CB. Project supervision and funding acquisition: NT-S and CB. All authors have read and agreed to the published version of the manuscript.

## Funding

The work reported in this publication was supported by the National Cancer Institute of the National Institutes of Health under the awards for the Partnership of Native American Cancer Prevention U54CA143924 (UACC) and U54CA143925 (NAU) and by the Centers for Disease Control and Prevention in a cooperative agreement with the Navajo Nation NU58DP006311.

## Conflict of Interest

The authors declare that the research was conducted in the absence of any commercial or financial relationships that could be construed as a potential conflict of interest.

## Publisher's Note

All claims expressed in this article are solely those of the authors and do not necessarily represent those of their affiliated organizations, or those of the publisher, the editors and the reviewers. Any product that may be evaluated in this article, or claim that may be made by its manufacturer, is not guaranteed or endorsed by the publisher.
